# Group A *Streptococcus* Secreted Esterase Hydrolyzes Platelet-Activating Factor to Impede Neutrophil Recruitment and Facilitate Innate Immune Evasion

**DOI:** 10.1371/journal.ppat.1002624

**Published:** 2012-04-05

**Authors:** Mengyao Liu, Hui Zhu, Jinquan Li, Cristiana C. Garcia, Wenchao Feng, Liliya N. Kirpotina, Jonathan Hilmer, Luciana P. Tavares, Arthur W. Layton, Mark T. Quinn, Brian Bothner, Mauro M. Teixeira, Benfang Lei

**Affiliations:** 1 Department of Immunology and Infectious Diseases, Montana State University, Bozeman, Montana, United States of America; 2 Department of Physiology, Harbin Medical University, Harbin, People's Republic of China; 3 State Key Laboratory of Agricultural Microbiology, Huazhong Agricultural University, Wuhan, People's Republic of China; 4 Laboratory of Immunopharmacology, Federal University of Minas Gerais, Belo Horizonte, Brazil; 5 Department of Chemistry and Biochemistry, Montana State University, Bozeman, Montana, United States of America; 6 Montana Veterinary Diagnostic Laboratory, Bozeman, Montana, United States of America; Children's Hospital Boston, United States of America

## Abstract

The innate immune system is the first line of host defense against invading organisms. Thus, pathogens have developed virulence mechanisms to evade the innate immune system. Here, we report a novel means for inhibition of neutrophil recruitment by Group A *Streptococcus* (GAS). Deletion of the secreted esterase gene (designated *sse*) in M1T1 GAS strains with (MGAS5005) and without (MGAS2221) a null *covS* mutation enhances neutrophil ingress to infection sites in the skin of mice. In trans expression of SsE in MGAS2221 reduces neutrophil recruitment and enhances skin invasion. The *sse* deletion mutant of MGAS5005 (Δ*sse* ^MGAS5005^) is more efficiently cleared from skin than the parent strain. SsE hydrolyzes the sn-2 ester bond of platelet-activating factor (PAF), converting biologically active PAF into inactive lyso-PAF. K_M_ and *k*_cat_ of SsE for hydrolysis of 2-thio-PAF were similar to those of the human plasma PAF acetylhydrolase. Treatment of PAF with SsE abolishes the capacity of PAF to induce activation and chemotaxis of human neutrophils. More importantly, PAF receptor-deficient mice significantly reduce neutrophil infiltration to the site of Δ*sse* ^MGAS5005^ infection. These findings identify the first secreted PAF acetylhydrolase of bacterial pathogens and support a novel GAS evasion mechanism that reduces phagocyte recruitment to sites of infection by inactivating PAF, providing a new paradigm for bacterial evasion of neutrophil responses.

## Author Summary

GAS is a major human pathogen causing a variety of infections, including pharyngitis and necrotizing fasciitis. GAS pathogenesis is mediated by a large array of secreted and cell-surface virulence factors. However, the functions of many GAS virulence factors are poorly understood. Recently, we reported that the esterase secreted by GAS (SsE) is a CovRS (the control of virulence two component regulatory system)-regulated protective antigen and is critical for spreading in the skin and systemic dissemination of GAS in a mouse model of necrotizing fasciitis. This report presents three major findings regarding the function and functional mechanism of SsE: 1) SsE contributes to GAS inhibition of neutrophil recruitment; 2) SsE is a potent PAF acetylhydrolase and the first secreted bacterial PAF acetylhydrolase identified so far; and 3) the PAF receptor significantly contributes to neutrophil recruitment in skin GAS infection. These findings support a novel mechanism for evasion of the innate immune system by GAS that may be relevant to other infections.

## Introduction

Neutrophils are one of the first responders of innate inflammatory cells to migrate towards the site of infecting agents. Evasion of the neutrophil microbicidal response is critical for survival, dissemination, and infectability of bacterial pathogens. Bacterial pathogens evade the neutrophil responses by multiple mechanisms, including inhibition of neutrophil infiltration, antiphagocytosis, and killing of neutrophils. Group A *Streptococcus* (GAS) causes a variety of diseases, ranging from relatively mild pharyngitis to potentially lethal invasive infections, such as necrotizing fasciitis [1]. The success of GAS as a pathogen is based, in part, on its ability to evade the innate immune system. GAS expresses extracellular peptidases ScpA and SpyCEP/ScpC to inhibit neutrophil recruitment by degrading the chemotactic C5a peptide and IL-8/CXC chemokines, respectively [2], [3], [4], [5]. The hyaluronic acid capsule and surface M protein made by GAS confer resistance to opsonophagocytosis and phagocytosis by neutrophils [6], [7]. Secreted DNase Sda1 helps GAS escape from neutrophil extracellular traps [8]. Mac/IdE inhibits opsonophagocytosis [9], [10]. Streptolysin S and streptolysin O kill and induce apoptosis of neutrophils [11], [12].

GAS pathogenesis is mediated by many virulence factors, and alteration in regulation of virulence factors greatly affects clinical outcomes. The two component regulatory system CsrRS/CovRS negatively regulates many virulence factor genes of GAS, including most of the virulence factors involved in the innate immune evasion [13], [14]. Nonsense and missense mutations in *csrRS*/*covRS* occur during human infections and are epidemiologically linked to severe GAS infections [15]. Selection of hypervirulent strains with *csrRS*/*covRS* mutations during experimental invasive infections in mice further highlights the critical role of *csrRS/covRS* mutations in progression of invasive GAS infections [16], [17], [18]. Loss of SpeB and enhanced production of the hyaluronic acid capsule contribute to the progression of invasive GAS infections [19], [20]. Enhanced production of the virulence factors in the innate immune evasion as a result of *csrRS*/*covRS* mutations plays a key role in selection for hypervirulent *csrRS*/*covRS* mutants. The DNase Sda1 helps GAS escape neutrophil extracellular traps and provides selection pressure for *csrRS*/*covRS* mutations [8]. Neutrophil infiltration to infection sites is almost completely inhibited in some necrotizing fasciitis patients and during experimental severe soft tissue infections in primates and mice [2], [21], [22], [23]. Enhanced production of SpyCEP/ScpC and ScpA as a result of *csrRS*/*covRS* mutations are believed to contribute to the enhanced inhibition of neutrophil recruitment in severe invasive infections.

It is not known whether SpyCEP/ScpC and ScpA are entirely responsible for the dramatic inhibition of neutrophil recruitment by hypervirulent GAS strains with *csrRS*/*covRS* mutations. Platelet-activating factor (PAF) also has chemotactic activity for inflammatory cells. PAF is a phospholipid mediator with the chemical structure of 1-O-alkyl-2-acetyl-sn-glycero-3-phosphorylcholine [24]. PAF is produced by endothelial cells, neutrophils, macrophages, and eosinophils in responses to proinflammatory cytokines, phagocytosis, and/or other stimuli [25]. This important phospholipid mediator has diverse and potent biological activities, including participation in normal physiological processes, such as inflammation, hemostasis, and reproduction, and contribution to pathological responses, including asthma, ischemia, gastric and pulmonary distress, allergy, and shock [26]. Particularly, PAF can activate platelets [27] and neutrophils [28]. The biological activities of PAF are mediated by a G protein-linked receptor (PAFR) that is expressed on the surface of various cell types [29], [30].

The biological activities of PAF are regulated by PAF acetylhydrolases that hydrolyze the sn-2 acetyl ester bond, converting PAF into acetic acid and lyso-PAF. Four mammal PAF acetylhydrolases, secreted or plasma, two intracellular type I, and intracellular type II PAF acetylhydrolases, have been described [31], [32], [33], [34]. The plasma and intracellular type II PAF acetylhydrolases belong to group VII of phospholipases A_2_, and the type I PAF acetylhydrolases are classified as group VIII phospholipases A2 [35]. Group VIII PAF acetylhydrolases are completely specific for PAF whereas the plasma and type II PAF acetylhydrolases hydrolyze unmodified sn-2 fatty acyl residues up to 5 or 6 carbon atoms long and longer sn-2 acyl residues with modification by oxidation [35]. PAF acetylhydrolase activity has been also detected in bacteria and fungus. An intracellular yeast group VII PAF acetylhydrolase enhances the viability of yeast under oxidative stress [36]. The spirochete *Leptospira interrogans* produces a PAF acetylhydrolase [37]. An apparently intracellular esterase Est13 from an earthworm gut-associated microorganism inhibits PAF-induced platelet aggregation [38]. Both *L. interrogans* PAF acetylhydrolase and Est13 share sequence homology with the α1 subunit of the type intracellular I mammalian PAF acetylhydrolase. The function of these bacterial PAF acetylhydrolases is not known. These yeast and bacterial PAF acetylhydrolases are intracellular proteins.

The esterase secreted by GAS (designated SsE) is a protective antigen [39] and is regulated by CsrRS/CovRS and required for GAS virulence and dissemination [40]. The basis for the contribution of SsE to GAS virulence and dissemination is unknown. Identification of the esterase target is essential for elucidating the functional mechanism of SsE. The homologue of SsE in the horse pathogen *Streptococcus equi* possesses optimal activity to acetyl esters [41]. We hypothesize that SsE targets PAF and is involved in evasion of the innate immune system. Here, we report on studies designed to test this hypothesis. Our findings demonstrate that SsE is indeed a potent PAF acetylhydrolase and is required for inhibition of neutrophil infiltration. We also present evidence for one of mechanisms for SsE to evade the neutrophil response by targeting PAF, identifying a new novel virulence factor for innate immune evasion.

## Results

### PAF Acetylhydrolase Activity of SsE

**Figure 1:**
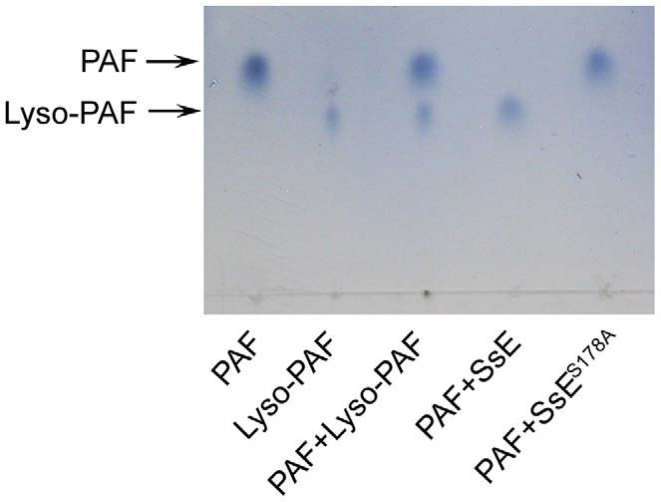
Demonstration of SsE-catalyzed conversion of PAF into lyso-PAF by TLC analysis. PAF was incubated with 80 nM SsE or SsE^S178A^ for 10 min, and 1 µl of the reaction solution was analyzed by TLC, as described under Materials and Methods. Authentic PAF, lyso-PAF, and their mixture were included to verify their rates of migration.

Identification of the esterase target is essential for elucidating the mechanism by which GAS uses SsE to contribute to GAS virulence and dissemination. Since the homologue of SsE in *Streptococcus equi* has optimal activities to acetyl esters [41], we considered whether the target of SsE is a molecule with a short-chain acyl ester group. PAF appears to be a good candidate as the target of SsE since it has an acetyl group and is an inflammatory mediator and chemoattractant [28], [42]. PAF was incubated with SsE, and the reaction was analyzed by thin layer chromatography (TLC), which could resolve PAF and lyso-PAF because PAF migrates much faster (Figure 1). SsE-treated PAF migrated the same distance as lyso-PAF, indicating that PAF was hydrolyzed by SsE. To confirm that PAF hydrolysis was due to the enzymatic activity of SsE, we performed a control experiment using SsE^S178A^ mutant protein. This mutant lacks the catalytic residue, Ser178, and, therefore, lacks enzymatic activity [39]. Indeed, SsE^S178A^-treated PAF and untreated PAF had the same migration rate. These results indicate that SsE hydrolyzes the acetyl ester bond in PAF, resulting in lyso-PAF.

**Figure 2:**
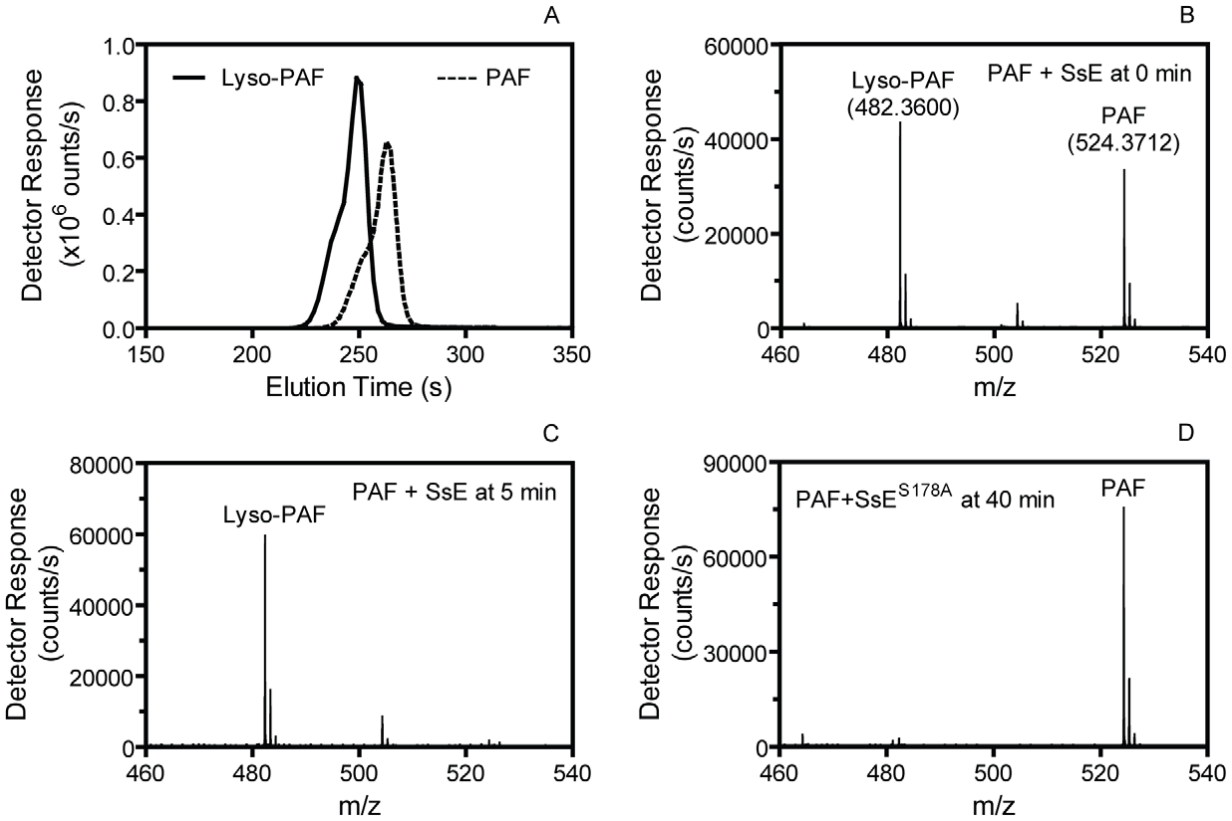
Confirmation of SsE-catalyzed hydrolysis of PAF. (A) HPLC elution profiles of PAF and lyso-PAF from a 1.4 mM PAF/80 nM SsE reaction sample taken at 0 min after mixing. (B) The average mass spectrum of the 0-min sample covering elution times from 3 to 5 min, showing a mixture of PAF and lyso-PAF. (C) The average mass spectrum of the PAF/SsE reaction sample taken at 5 min after mixing covering elution times from 3 to 5 min. (D) The average mass spectrum of a 1.4 mM PAF/80 nM SsE^S178A^ reaction sample taken at 40 min after mixing.

Next, we used liquid chromatography/positive ion electrospray mass spectrometry (LC-MS) to confirm SsE-catalyzed hydrolysis of PAF. PAF (1.4 mM) was mixed with 80 nM SsE, and an aliquot was taken from the reaction immediately (0 min) or at 5 min after mixing and diluted with an equal volume of acetonitrile to stop the reaction. A control reaction containing PAF and SsE^S178A^ was performed under the identical conditions and stopped at 40 min after mixing. The elution times of lyso-PAF and PAF on a C8 column were 4.15 and 4.38 min, respectively (Figure 2A), and the accurate m/z values of PAF and lyso-PAF were 524.3722 and 482.3600, respectively (Figure 2B). We found that 57% and 100% of PAF was converted into lyso-PAF for the SsE-treated PAF samples obtained at 0 and 5 min after mixing, respectively (Figure 2B and 2C), whereas no PAF was hydrolyzed into lyso-PAF at 40 min after mixing PAF with inactive SsE^S178A^ (Figure 2D). These results unambiguously demonstrate that SsE catalyzes the conversion of PAF into lyso-PAF. Thus, SsE is a PAF acetylhydrolase.

**Figure 3:**
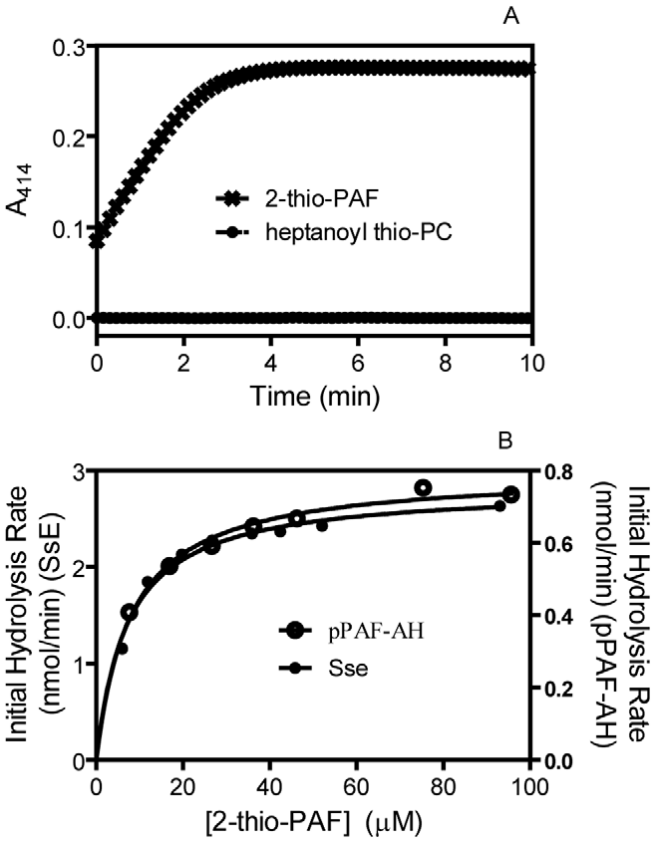
SsE hydrolyzes 2-thio-PAF but not heptanoyl thio-PC. (A) Time course of absorbance change at A_414_ after mixing 3.4 nM SsE with 40 µM 2-thio-PAF or 30 nM SsE with 40 µM heptanoyl thio-PC. (B) Initial rates of SsE-catalyzed hydrolysis of 2-thio-PAF as a function of corrected 2-thio-PAF concentration. The rates were calculated from the slopes in panel A of Figure S1 using 0.68 pmole SsE, and the correction of [2-thio-PAF] is described in Figure S1. The rates of 2-thio-PAF hydrolysis using 0.86 pmole human plasma PAF acetylhydrolase are also presented as a comparison.

To determine whether SsE can hydrolyze long-chain acyl group at the sn-2 position, we tested whether SsE hydrolyzes heptanoyl thio-PC (1-O-hexadecyl-2-heptanoyl glycerol-3-phosphocholine), an analogue of 2-thio-PAF, which is used in a colorimetric assay for PAF acetylhydrolases [43]. No hydrolysis of heptanoyl thio-PC was detected, whereas 2-thio-PAF was rapidly hydrolyzed (Figure 3A), indicating that SsE cannot hydrolyze esters with a long-chain acyl group. We also determined whether the PAF acetylhydrolase activity of SsE requires Ca^2+^. The observed initial hydrolysis rates were measured in reactions containing 2.3 nM SsE and 20 µM 2-thio-PAF in the presence of 0.0 mM Ca^2+^, 1.0 mM EDTA, or 1.0 mM Ca^2+^. The measured rates of 2-thio-PAF hydrolysis were 7.0, 9.2, and 7.8 µM min^−1^, respectively. Thus, the activity of SsE does not require Ca^2+^ and other metal ions that can form a complex with EDTA. These properties of SsE are similar to those of eukaryotic PAF acetylhydrolases.

### Enzymatic Parameters of the PAF Acetylhydrolase Activity of SsE

To determine whether SsE is a potent PAF acetylhydrolase, we measured the *k*_cat_ and K_M_ values of SsE for hydrolysis of 2-thio-PAF using the PAF acetylhydrolase assay kit and compared them with those of recombinant human plasma PAF acetylhydrolase. The initial reaction rates were obtained as described in Figure S1. The relationship of the observed rates versus 2-thio-PAF concentration fits the Michaelis-Menten equation (Figure 3B), yielding a *k*_cat_ of 69.6 s^−1^ and an apparent K_M_ of 7.0 µM for SsE. In comparison, *k*_cat_ and K_M_ of recombinant human plasma PAF acetylhydrolase were determined to be 15.4 s^−1^ and 8.0 µM, respectively. These measurements indicate that SsE has similar K_M_ with and higher *k*_cat_ than the human enzyme.

### Inhibition of PAF-Induced Activation and Chemotaxis of Human Neutrophils by SsE

**Figure 4:**
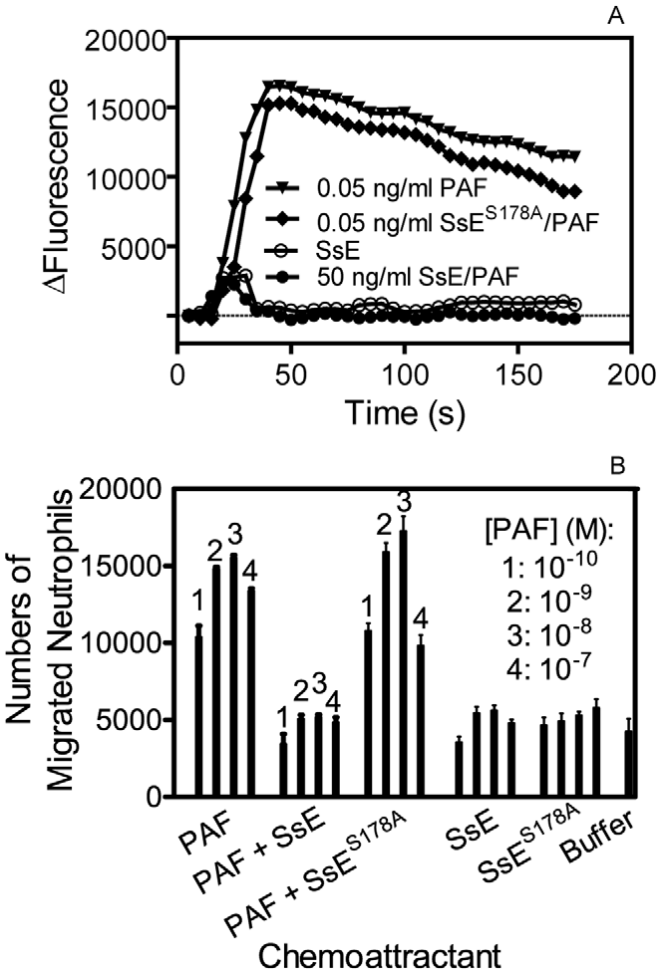
SsE abolishes PAF-induced activation and chemotaxis of neutrophils. (A) SsE treatment of PAF abolishes PAF-induced Ca^2+^ mobilization in human neutrophils. Neutrophils (2×10^5^ cells/well) loaded with Fluo-4 acetoxymethyl ester dye were mixed with 0.05 ng/ml PAF, 0.05 ng/ml SsE^S178A^-treated PAF, 50 ng/ml SsE-treated PAF, or SsE alone control, and Ca^2+^ flux was recorded. Presented are time courses of the fluorescence intensity after addition of PAF, SsE-treated PAF, or SsE or buffer controls. (B) Number of neutrophils migrated to PAF, SsE- and SsE^S178A^-treated PAF, SsE, SsE^S178A^, and buffer in a transwell cell migration assay [58] (See the Methods section for experimental details).

PAF has a variety of biological functions, including activation of neutrophils, and the acetyl ester group at sn-2 is critical for its activities. Thus, SsE-catalyzed hydrolysis of PAF should inactivate the functions of PAF. We tested whether treatment of PAF with SsE alters the capacity of PAF to activate neutrophil Ca^2+^ mobilization. SsE-treated, SsE^S187A^-treated, and untreated PAF and SsE alone were added to human neutrophils preloaded with Fluo-4 acetoxymethyl ester, and changes in fluorescence due to the increase in free intracellular Ca^2+^ were monitored. SsE-treated PAF at 50 ng/ml and the protein controls were not able to mobilize an intracellular Ca^2+^ flux, whereas SsE^S178A^-treated and untreated PAF at 0.05 ng/ml induced a normal Ca^2+^ flux (Figure 4A). Thus, SsE abolishes the capacity of PAF to activate this neutrophil response.

PAF is also a potent neutrophil chemoattractant. To determine whether SsE could inhibit PAF-induced neutrophil chemotaxis, we assessed the effect of SsE on PAF-induced neutrophil migration. As shown in Figure 4B, PAF was chemotactic for human neutrophils, whereas SsE-treated PAF lost the chemotactic activity, and the number of migrated neutrophils in the presence of PAF that was treated with SsE were similar to those of the buffer and protein only controls. In contrast, treatment with inactive SsE^S178A^ did not reduce PAF-induced neutrophil chemotaxis (Figure 4B). These results indicate that SsE inhibits PAF-induced neutrophil chemotaxis and that the inhibition requires SsE enzymatic activity.

### Enhanced Neutrophil Ingress to Δ*sse* ^MGAS5005^ Infection Sites

**Figure 5:**
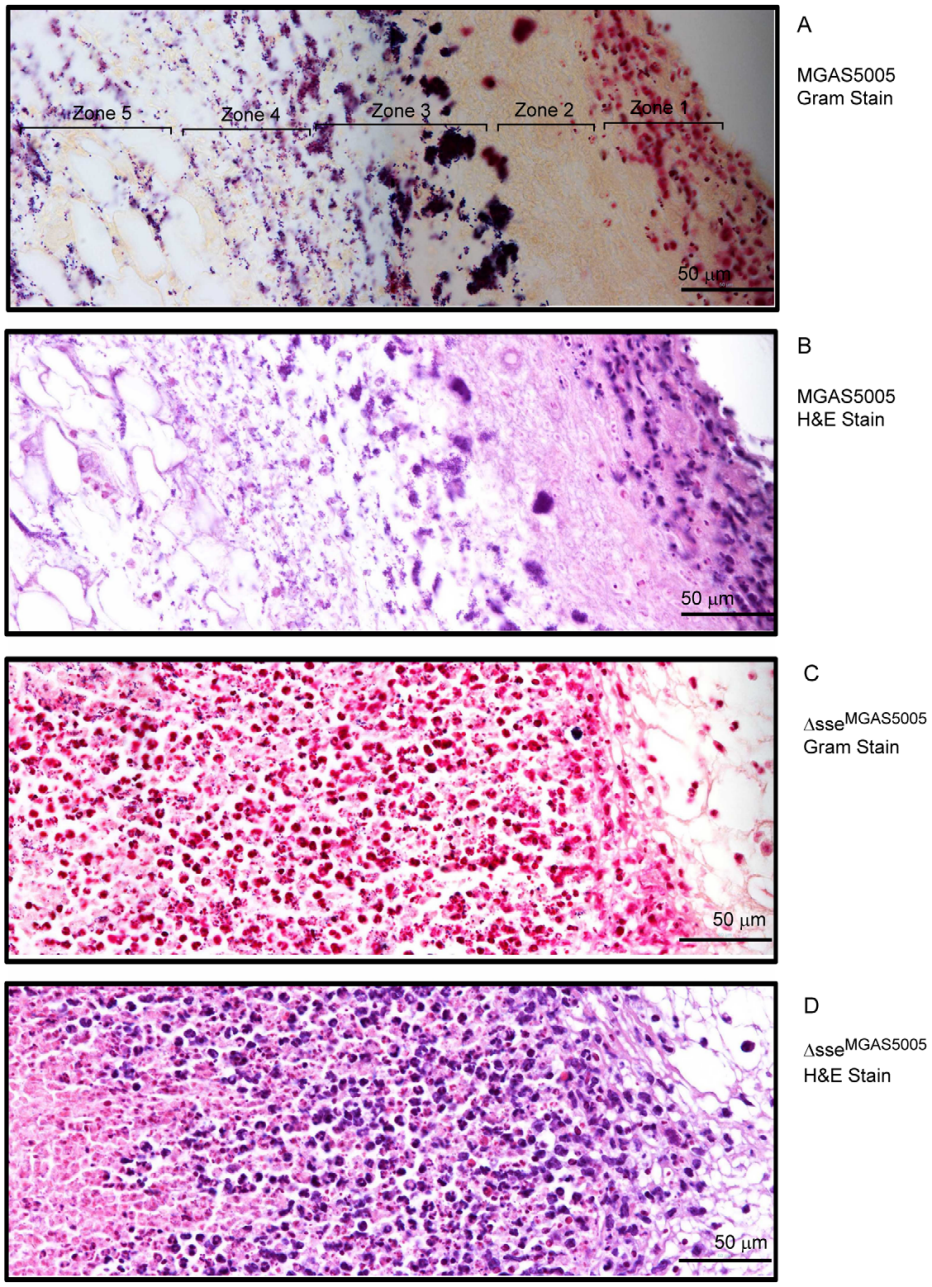
Histological analyses showing the difference in levels and patterns of inflammatory cell infiltration between the MGAS5005 and Δ*sse* ^MGAS5005^ infections. BALB/c mice were subcutaneously inoculated on the back with 1.0×10^8^ cfu MGAS5005 or 1.1×10^8^ cfu Δ*sse* ^MGAS5005^, and the skin samples were collected at 24 h after inoculation. The microscopic pictures of the Gram and H&E-stained samples were each combined from three snapshots that were taken at a 40× magnification. The five zones in panel A represent different morphologies. Panels: (A), MGAS5005/Gram stain; (B), MGAS5005/H&E Stain; (C), Δ*sse* ^MGAS5005^/Gram stain; and (D), Δ*sse* ^MGAS5005^/H&E stain.

Since SsE can abolish PAF-induced activation and chemotaxis of neutrophils, we tested whether SsE is involved in innate immune evasion during GAS infections. We first examined the infection sites and performed histological analyses. MGAS5005 extensively spreads from inoculation sites by 24 hours after inoculation (Figure S2A) whereas the Δ*sse* ^MGAS5005^ mutant remained at the inoculation site (Figure S2B). The histological analyses of the skin infection sites with the Gram and hematoxylin and eosin (H&E) stains reveal distinct patterns of inflammatory cell infiltration between MGAS5005 and Δ*sse* ^MGAS5005^ sites at 24 h after inoculation. Inflammatory cells and amorphous materials were kept away from GAS at the MGAS5005 inoculation site, and few neutrophils were found at the spread area of MGAS5005 (Figure S3A and S3B). In contrast, inflammatory cells were present throughout the inoculation site with more inflammatory cells surrounding the infection site (Figure S3C and S3D). The distinct details of these patterns are more evident at a higher magnification. There are five morphological zones at an end of the MGAS5005 inoculation site starting from the interior side of the skin (the right side in panels A and B of Figure 5): Zone 1, neutrophils and other inflammatory cells without GAS; Zone 2, amorphous host materials lack of GAS; Zone 3, a few inflammatory cells that could reach the boundary of the GAS territory were victimized by and associated with massive amount of GAS; Zone 4, necrotized adipose tissue and GAS without inflammatory cells; and Zone 5, invasion of GAS along the interstitial space of the adipose cells (Figure 5A and 5B). Thus, MGAS5005 not only reduces infiltration of neutrophils but also keep inflammatory cells away. However, inflammatory cells and Δ*sse* ^MGAS5005^ bacteria were mingled throughout the infection site (Figure 5C and 5D). Similar results were obtained in CD-1 Swiss mice.

**Figure 6:**
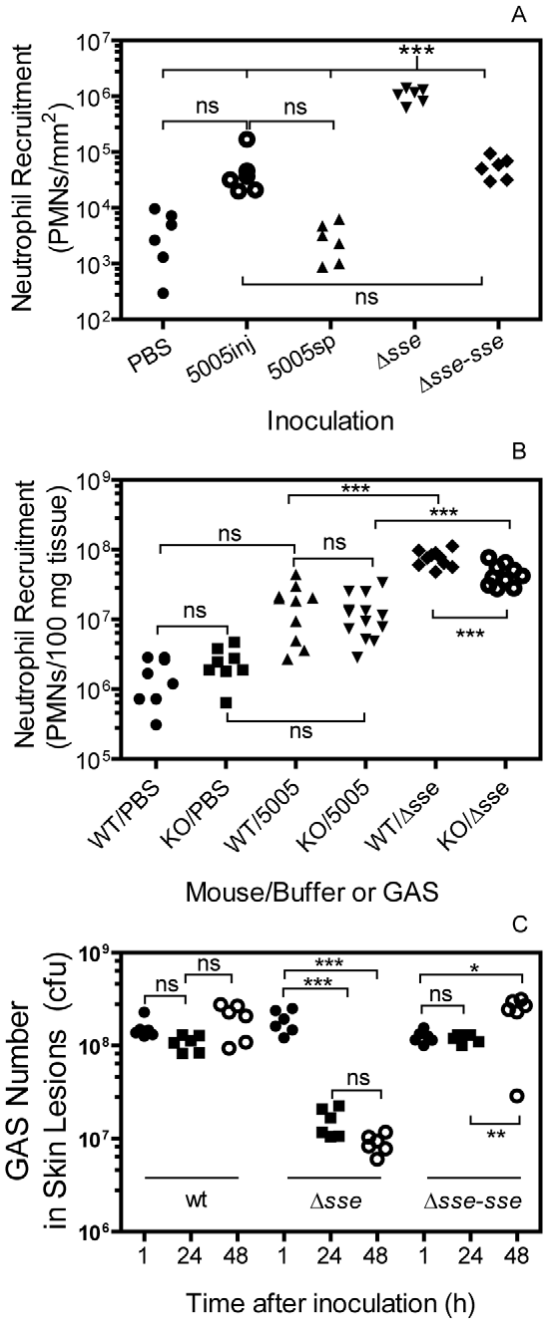
Evidence for the role of SsE in evasion of bactericidal neutrophil responses. (A) Deletion of *sse* enhances neutrophil recruitment. BALB/c mice were subcutaneously inoculated on the back with 1.0×10^8^ cfu MGAS5005 or 1.1×10^8^ Δ*sse* ^MGAS5005^. Neutrophil numbers at 24 h post-inoculation were determined by the myeloperoxidase assay. *** (One-way ANOVA analysis): The data is significant for Δ*sse* site versus buffer control (PBS), MGAS5005 inoculation site (5005inj), MGAS5005 spread area (5005sp), and reverse-complement strain of Δ*sse* (Δ*sse*-*sse*). (B) Significant reduction in neutrophil ingress to Δ*sse* site, but not to MGAS5005 site, in PAFR^−/−^ mice compared with BALB/c control mice. Five PAFR^−/−^ (KO) or BALB/c (wt control) mice per group were subcutaneously inoculated with 0.2 ml PBS, MGAS5005, or Δ*sse* ^MGAS5005^ suspension at OD_600_ of 0.8. The statistic analysis data (***, significant; ns, not significant) were obtained from one-way ANOVA analysis of the combined data of two experiments. (C) Efficient clearance of Δ*sse* ^MGAS5005^. Numbers of viable GAS at sites of MGAS5005 and Δ*sse* ^MGAS5005^ infection at 1, 24, and 48 h after inoculation are shown. Six mice were used for each time point and each strain. Inoculum: MGAS5005, 1.1×10^8^ cfu; Δ*sse*, 1.2×10^8^ cfu; Δ*sse-sse*, 1.0×10^8^ cfu.

Next, we used the myeloperoxidase assay [44] to quantify neutrophil ingress to the skin infection sites of MGAS5005 and Δ*sse* ^MGAS5005^ at 24 h after subcutaneous infection of BALB/c mice. The mean neutrophil number ± SD of the Δ*sse* infection site was (1.1±0.12)×10^6^/mm^2^, which was 19.6 and 346-fold greater than the neutrophil number at the MGAS5005 inoculation site [(5.4±2.3)×10^4^ neutrophils/mm^2^] and at the spread infection area of MGAS5005 [(3.1±0.87)×10^3^ neutrophils/mm^2^]. Reverse complementation of Δ*sse* ^MGAS5005^ with the *sse* gene (Δ*sse*-*sse*) restored the inhibition of neutrophil recruitment [(5.6±1.0)×10^4^ neutrophils/mm^2^]. The difference is significant between the Δ*sse* ^MGAS5005^ sample and each of the other samples but insignificant among the other samples in one way ANOVA analysis using the Tukey's Multiple Comparison Test (Figure 6A).

### Reduction of Neutrophil Ingress to Δ*sse* ^MGAS5005^ Sites in PAF Receptor-Deficient Mice

The receptor of PAF (PAFR) is a G protein-coupled receptor that mediates the biological activities of PAF. We used PAFR-deficient mice [30] to test whether SsE inhibits neutrophil infiltration by hydrolyzing PAF. MGAS5005 induced low and similar levels of neutrophil recruitment in both BALB/c and PAFR^−/−^ mice. However, the mean number of recruited neutrophils at the Δ*sse* ^MGAS5005^ infection site was reduced by 47% in PAFR^−/−^ mice compared with BALB/c mice (Figure 6B). The reduction of neutrophil influx due to the absence of the PAF receptor was 52.7% of the enhancement of neutrophil influx as a result of the *sse* deletion. These results suggest that targeting PAF by SsE is an equally important mechanism as an PAF-independent mechanism. These results strongly suggest that PAF plays a significant role in neutrophil infiltration in GAS infections and that SsE-mediated hydrolysis of PAF contributes to the observed reduction in neutrophil infiltration.

### Efficient Clearance of Δ*sse* ^MGAS5005^ by Neutrophils

Since Δ*sse* bacteria were associated with high levels of neutrophils, these bacteria should be killed by recruited neutrophils. Indeed, the numbers of viable Δ*sse* ^MGAS5005^ at 24 and 48 hours post-inoculation were 8.3% and 4.8% of those found at 1 h after inoculation, respectively; whereas the numbers of MGAS5005 at 24 and 48 h post-inoculation were 70% and 128% of those found at 1 h after inoculation, respectively (Figure 6C), suggesting that Δ*sse* ^MGAS5005^ is cleared more efficiently than MGAS5005 at skin infection sites.

### No Detrimental Effects of *sse* Deletion on Transcription of *spyCEP*, *scpA*, and Other CsrRS/CovRS- and Mga-Regulated Genes

In a transcription profiling analysis for MGAS5005 and Δ*sse* ^MGAS5005^ using the NimbleExpress *Streptococcus pyogenes* arrays, the transcription levels of the genes regulated by the multiple gene regulator of GAS (Mga) and CsrRS/CovRS in Δ*sse* ^MGAS5005^ were 70% to 135% of those in MGAS5005 at the mid-exponential growth phase except that *sse* transcript was not detected in Δ*sse* ^MGAS5005^ (Figure S4). These results rule out the possibility that the phenotype of Δ*sse* ^MGAS5005^ is caused by alteration in transcription of the *scpA*, *spyCEP/scpC*, *sda1*/*sdaD2*, *slo*, *sagA*, *hasA*, *speB*, and *emm* genes, which are involved in innate immune evasion by GAS.

### Effects of *sse* Deletion on Virulence, Soft Tissue Invasion, and Neutrophil Recruitment in MGAS2221 Infection

**Figure 7:**
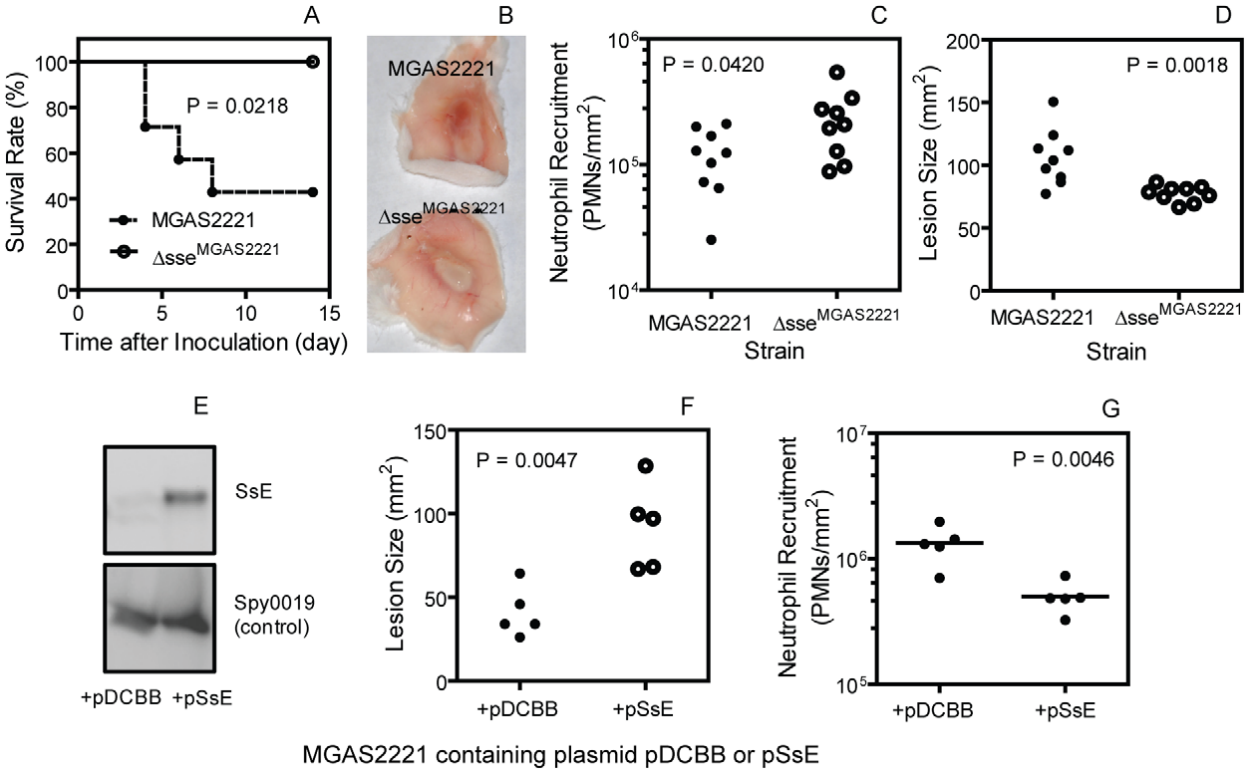
Effects of *sse* deletion and in trans overexpression on virulence, neutrophil recruitment, and skin invasion in MGAS2221 infection. (A) Survival rates of BALB/c mice infected subcutaneously with 1.5×10^8^ cfu MGAS2221 or Δ*sse* ^MGAS2221^. (B–D) Inside-out infection site (B), neutrophil recruitment (C), and lesion size (D) of BALB/c mice at 24 h after subcutaneous inoculation with 3.9×10^7^ cfu MGAS2221 or 1.6×10^8^ cfu Δ*sse* ^MGAS2221^. (E) Western blots showing overproduction of SsE in the culture supernatant of MGAS2221 containing *sse* gene-containing pSsE. MGAS2221 containing pDCBB was a vector control, and Spy0019 was a secreted protein control. (F and G) Lesion size (F) and neutrophil recruitment (G) in mice infected with 9.2×10^6^ cfu MGAS2221/pDCBB (+pDCBB) or 9.3×10^6^ cfu MGAS2221/pSsE (+pSsE).

MGAS5005 has a natural null *covS* deletion, which enhances expression of *sse* and many other virulence genes [18], [40]. To test whether SsE contributes to pathogenesis and inhibition of neutrophil recruitment in GAS with the wild-type *csrRS/covRS* genes, we deleted the *sse* gene in MGAS2221. Fifty seven percent of BALB/c mice infected subcutaneously with 1.5×10^8^ cfu of MGAS2221 were dead whereas all mice infected with 1.6×10^8^ cfu Δ*sse* ^MGAS2221^ survived (P = 0.0218) (Figure 7A). In a separate experiment, 3.9×10^7^ cfu MGAS2221 or 1.6×10^8^ cfu Δ*sse* ^MGAS2221^ bacteria were inoculated into BALB/c mice. The lesion appearance was obviously different between the wt and mutant infection sites (Figure 7B). The number of neutrophils at the Δ*sse* ^MGAS2221^ site was significantly higher than that at the MGAS2221 site (mean neutrophil number ± SD: Δ*sse* ^MGAS2221^, (2.4±1.4)×10^5^/mm^2^; MGAS2221, (1.2±0.6)×10^5^/mm^2^) (P = 0.0420) (Figure 7C). Conversely, the size of the Δ*sse* ^MGAS2221^ site was significant smaller than that of the MGAS2221 site (mean size ± SD: MGAS2221, 106±20 mm^2^; Δ*sse* ^MGAS2221^, 77±6 mm^2^) (P = 0.0014) (Figure 7D). It should be stressed that the significant role of SsE in the invasion of skin tissue and inhibition of neutrophil recruitment was observed with a dose of Δ*sse* ^MGAS2221^ that was 3 times higher than that of MGAS2221. The results using the higher dose of the mutant suggest that the mutant phenotype is not caused by a growth defect. Thus, SsE can reduce neutrophil recruitment and enhances soft tissue invasion in infection with a representative M1T1 strain with the wild-type *csrRS/covRS* background.

### Inhibition of Neutrophil Recruitment and Enhancement of Soft Tissue Invasion by In Trans Expression of SsE in MGAS2221

The effects of in trans expression of SsE on neutrophil recruitment and lesion size during subcutaneous MGAS2221 infection of mice further confirm the role of SsE in inhibition of neutrophil recruitment and enhancement of soft tissue invasion by GAS. The *sse* gene was cloned into pDCBB [45], yielding pSsE. At the early growth phase (OD_600_ = 0.2), SsE was detected in the supernatant of MGAS2221/pSsE but not MGAS2221/pDCBB (vector control) by Western blotting analysis, whereas the secreted protein Spy0019 was detected at similar levels in the supernatant of both strains (Figure 7E). These results indicate that the introduction of pSsE into MGAS2221 enhances SsE production. In trans production of SsE increased lesion size by 124% compared with the vector control (Lesion size ± SD: MGAS2221/pDCBB, 41±15 mm^2^; MGAS2221/pSsE, 92±25 mm^2^) (P = 0.0047) (Figure 7F). Inversely, in trans production of SsE reduced neutrophil recruitment by 72% (mean neutrophil number ± SD: MGAS2221/pDCBB, (6.2±0.28)×10^5^/mm^2^; MGAS2221/pSsE, 1.7±0.11)×10^5^/mm^2^) (P = 0.0111) (Figure 7G).

### *In Vitro* and *In Vivo* Growth of Δ*sse* ^MGAS5005^ and Δ*sse* ^MGAS2221^

**Figure 8:**
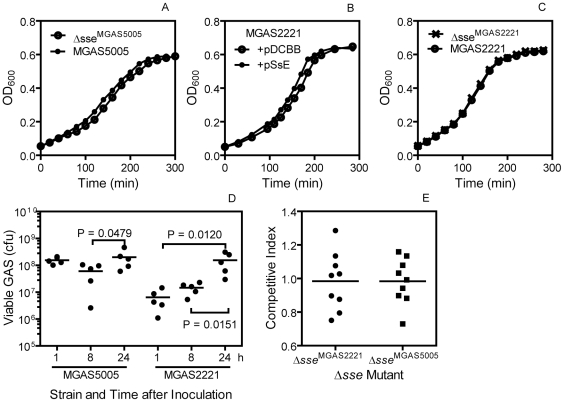
*In vitro* and *in vivo* growth of GAS strain with *sse* deletion or in trans expression. (A–C) Growth curve of MGAS5005 and Δ*sse* ^MGAS5005^ (A), MGAS2221/pDCBB and MGAS2221/pSsE (B), and MGAS2221 and Δ*sse* ^MGAS2221^ (C) in THY. Each culture at the mid-exponential growth phase was diluted at time zero to start measurement of OD_600_ with time. (D) Numbers of MGAS2221 and MGAS5005 in an air sac at 1, 8 and 24 h after subcutaneous inoculation of 1.4×10^8^ cfu MGAS2221 or 1.3×10^8^ cfu MGAS5005 in 0.1 ml PBS with 0.9 ml air. (E) Competitive growth index of Δ*sse* ^MGAS2221^ and Δ*sse* ^MGAS5005^ against MGAS2221 and MGAS5005, respectively, at 24 h after inoculation in the model of air sac subcutaneous infection.

The Δ*sse* ^MGAS5005^ mutant has a longer early growth phase by about 15 min (Figure 8A) and about 10% more viable CFU per OD_600_ at the exponential growth phase (data not shown) than its parent strain in Todd-Hewitt broth supplemented with 0.2% yeast extract (THY). Consistent with this result, in trans overexpression of SsE in MGAS2221 shows a 20-min shorter early growth phase than the vector control (Figure 8B). However, MGAS2221 and Δ*sse* ^MGAS2221^ have identical growth curves in THY (Figure 8C). Thus, the effect of *sse* expression on the length of early growth phase is obvious when SsE is highly produced.

To examine the growth of the mutants *in vivo*, we performed a competitive growth assay using an air sac infection model. A 1∶1 Δ*sse* ^MGAS2221^∶MGAS2221 or Δ*sse* ^MGAS5005^∶MGAS5005 mixture was injected with air in the subcutis of mice, and, 24 h later, the air sac was lavaged after the mice were euthanized. The lavage samples were plated, and the Δ*sse*∶wt GAS ratio of the GAS colonies was determined by PCR analysis. The Δ*sse*∶wt GAS ratio in the inoculum was measured by plating the individual GAS suspension prior to mixing. The mean number of MGAS5005 and MGAS2221 at 24 h was 11 and 3 times as the corresponding number at 8 h, respectively (Figure 8D), indicating that GAS grew in the air sac. The competitive index, the Δ*sse*∶wt ratio in the lavage sample/the ratio in the inoculum, for both Δ*sse* ^MGAS2221^ and Δ*sse* ^MGAS5005^ has a mean value of about 1 (Figure 8E), indicating that each mutant and its parent strain have similar growth *in vivo*. These data indicate that the phenotype of Δ*sse* ^MGAS5005^ and Δ*sse* ^MGAS2221^ is not caused by a growth phenotype.

## Discussion

This study presents three major findings regarding evasion of the innate immune system by GAS. First, SsE significantly contributes to GAS inhibition of neutrophil recruitment. Second, SsE is a potent PAF acetylhydrolase and the first secreted bacterial PAF acetylhydrolase identified so far. Third, SsE inactivates the ability of PAF to induce activation and migration of neutrophils, and the PAF receptor significantly contributes to neutrophil recruitment in skin GAS infection. These findings identify a new means for evasion of the innate immune system by GAS and support a novel paradigm for bacterial inhibition of neutrophil recruitment and function in which neutrophil recruitment is reduced by inactivating PAF.

One conclusion of this work is that SsE is required for the severe inhibition of neutrophil recruitment by MGAS5005 in the mouse model of necrotizing fasciitis. This nearly complete inhibition of neutrophil infiltration is similar to that of severe GAS infections in some human patients and experimental animal infections [2], [21], [22], [23]. In addition, SsE is critical for the virulence and dissemination of MGAS5005 and is a protective antigen [39], [40]. SsE also reduces neutrophil recruitment and enhances virulence and skin tissue invasion in infection with MGAS2221. Thus, SsE is a significant contributor to the innate immune evasion and tissue invasion by GAS with or without *covRS* mutations. It is well known that GAS produces C5a peptidase ScpA and IL-8/CXC peptidase SpyCEP/ScpC to reduce neutrophil recruitment. SpyCEP/ScpC reduces neutrophil infiltration in soft tissue infections of mice [2], [3], promotes resistance to neutrophil killing [5] and GAS dissemination [46], [47], and alters pathogenesis [48]. Immunization with ScpA prevents nasopharyngeal GAS colonization of mice [49]. GAS also produces virulence factors, such as the hyaluronic acid capsule, M protein, streptolysins S and O, opsonophagocytosis inhibitor Mac/IdeS, and DNases, to cripple the innate immune system. Our work adds a new virulence factor to the large array of GAS virulence factors that interfere with the bactericidal function of neutrophils.

Another conclusion of this work is that SsE contributes to the enhanced inhibition of neutrophil recruitment as a result of the null *covS* mutation in MGAS5005. MGAS5005 has a genetic makeup almost identical with that of MGAS2221, displaying 7 synonymous and 9 non-synonymous single nucleotide alterations, two single base mutation, and presence of an IS element [18]. However, MGAS5005 has a null *covS* deletion but MGAS2221 has the wild-type *csrRS/covRS* genes. Alteration of the transcription of the CsrRS/CovRS-regulated genes by the null *covS* mutation is apparently the cause for the lower neutrophil recruitment in MGAS5005 infection than in MGAS2221 infection. Expression of the *sse* gene is enhanced by 30 fold by the *covS* null mutation in MGAS5005 [40]. Deletion of the *sse* gene in MGAS5005 does not dramatically change expression of the CsrRS/CovRS- and Mga-regulated virulence genes but reversed the *covS* mutation-induced reduction of neutrophil infiltration. Thus, the enhanced production of SsE is an critical factor for the increase in inhibition of neutrophil recruitment and soft tissue infection during MGAS5005 infection in comparison with MGAS2221 infection. This conclusion is supported by the decrease in neutrophil recruitment and increase in skin invasion that are caused by in trans production of SsE in MGAS2221. SpyCEP/ScpC and ScpA are also up-regulated as a result of *covS* null mutations [45]. We propose that SsE, SpyCEP/ScpC, and ScpA can all reduce neutrophil recruitment during infections of GAS with the wild-type *covRS* genes and cripple neutrophil infiltration when they are highly produced as a result of null *covS* mutations. The requirement of SsE in the inhibition of neutrophil infiltration and invasion of skin tissue by MGAS5005 indicates that enhanced production of SsE, like enhancement in capsule production and suppression of SpeB production, is critical for *covS* mutations-mediated progression of invasive GAS infection.

SsE is a novel, potent bacterial PAF acetylhydrolase. Hydrolysis of PAF by SsE was clearly demonstrated by TLC. Analysis of the SsE/PAF reaction by LC-MS not only confirmed PAF hydrolysis but also demonstrated that SsE-catalyzed PAF hydrolysis was rapid. The PAF acetylhydrolase activity of SsE appears at least to be as potent as the human plasma PAF acetylhydrolase. PAF acetylhydrolase activity has also been detected in bacteria and yeast [36], [37], [38]. While the yeast PAF acetylhydrolase enhances the viability of yeast under oxidative stress, the function of *L. interrogans* PAF acetylhydrolase and Est13 is not known. There is a difference between SsE and the yeast, *L. interrogans*, and Est13 PAF acetylhydrolases in cellular location. SsE is a secreted protein [39] but the fungus and other bacterial PAF acetylhydrolases described so far are intracellular proteins [36]–[38]. This difference in the cellular location dictates whether these PAF acetylhydrolases can target host PAF. SsE would be able to degrade host PAF produced in response to infection but the fungus and other bacterial PAF acetylhydrolases should not be able to target host PAF. Thus, we have identified the first secreted PAF acetylhydrolase that can target host PAF. SsE has homologues in both Gram-positive and Gram-negative pathogens, such as *Streptococcus agalactiae*, *Streptococcus equi*, *Streptococcus zooepidemicus*, *Staphylococcus aureus*, *Streptobacillus moniliformis*, and *Actinomyces coleocanis* [41,BLAST results not shown]. The function and functional mechanism of SsE may be relevant to other bacterial infections.

Neutrophil infiltration is significantly reduced in Δ*sse* ^MGAS5005^ but not in MGAS5005 infection in the PAFR^−/−^ mice compared with those in the control mice. These results support a novel mechanism of innate immune evasion: SsE hydrolyzes PAF to reduce neutrophil recruitment. The reduction of neutrophil recruitment to Δ*sse* ^MGAS5005^ in PAFR^−/−^ is 52% of the enhancement of neutrophil recruitment caused by the *sse* deletion in MGAS5005. Thus, the PAF-dependent mechanism is a significant but not only mechanism for SsE to contribute to inhibition of neutrophil recruitment.

The role of SsE in skin invasion and inhibition of neutrophil recruitment appears not to be caused by a growth phenotype. First, Δ*sse* ^MGAS2221^ and MGAS2221 have similar growth both *in vitro* and *in vivo*, and, thus, the phenotype of Δ*sse* ^MGAS2221^ in neutrophil recruitment, skin invasion and virulence is not caused by a growth phenotype. Furthermore, Δ*sse* ^MGAS2221^ at a dose 4 times higher than that of MGAS2221 displayed the Δ*sse* phenotype. Third, although Δ*sse* ^MGAS5005^ has a longer early growth phase than the parent strain, the two strains have similar growth *in vivo*. Fourth, the decrease in neutrophil recruitment during the infection of PAFR^−/−^ mice with Δ*sse* ^MGAS5005^ cannot be explained by a growth phenotype. Finally, immunization of mice with SsE reduces skin invasion by MGAS5005 [39]. Furthermore, the Δ*sse* phenotype is apparently caused by the loss of SsE but not through an indirect effect since the *sse* deletion did not alter the expression of CsrRS/CovRS- and Mga-regulated virulence factors.

Δ*sse* ^MGAS5005^ has lower cfu numbers than that of MGAS5005 after 4-h incubation in serum [40]. However, this difference in growth in serum between MGAS5005 and Δ*sse* ^MGAS5005^ is not reflected in the air sac competitive growth assay. The different results in the two assays could depend on the effect of SsE on the early growth phase. High levels of SsE production as a result of *covS* mutation or in trans overexpression apparently shorten the early growth phase but did not change the doubling time *in vitro*. The effect of SsE on the length of the early growth phase might be the reason for the difference in cfu of Δ*sse* ^MGAS5005^ and MGAS5005 in serum because a low dose of bacteria (10^5^ cfu) were inoculated in the serum growth assay. The early growth phase of Δ*sse* ^MGAS5005^ in the air sac assay may be shortened because nearly 1,000-fold more Δ*sse* ^MGAS5005^ was inoculated in the air sac assay. At the same time, the early growth phase of MGAS5005 in the air sac assay could be longer than that in the serum growth assay because the nutrient in the air sac assay should be less abundant than in serum. Besides the effect on the early growth phase, the yield of Δ*sse* ^MGAS5005^ in chemically defined medium is lower than that of MGAS5005 [40], suggesting that SsE may be able to recycle metabolites or surface structures. These differential *in vitro* growth features of Δ*sse* ^MGAS5005^ and MGAS5005 appear not to be displayed *in vivo*, suggesting that the *in vitro* difference does not represent a genuine growth defect. Nonetheless, the *in vitro* growth data indicate that SsE can act on the GAS bacteria. This action could be the basis for a PAF-independent mechanism, in addition to the PAF-dependent mechanism, for the innate immune evasion by SsE.

Neutrophil influx to Δ*sse* ^MGAS5005^ sites in the PAFR^−/−^ mice was half of that in the control mice. This is the first demonstration for the importance of the PAF receptor in neutrophil recruitment in response to a bacterial infection. The PAF receptor is not critical for neutrophil infiltration in pulmonary *Klebsiella pneumonia*, *Pseudomonas aeruginosa*, *Streptococcus pneumoniae* infections and polymicrobial sepsis caused by cecum ligation and puncture [50], [51], [52], [53]. This difference suggests that PAF may play a critical role in neutrophil recruitment in skin infection but not in pulmonary infections. It is also possible that these pathogens, like MGAS5005, can inactivate PAF.

Hermoso et al. have found that the protein Pce of *Streptococcus pneumoniae* hydrolyzes the phosphocholine group of PAF and hypothesized that Pce has the capacity to interact with and hydrolyze PAF in the bloodstream *in vivo*, impacting on pathogenesis [54]. Apparently, bacterial pathogens have evolved different enzymatic activities to eliminate PAF, supporting an important role of PAF in host responses against bacterial infections.

PAF can be involved in innate immune responses in different ways. Administration of PAF can lead to neutrophil infiltration in the lung and skin [55], and PAF may participate in the inflammatory responses during GAS infections. IL-12-induced chemotaxis of NK cells and neutrophils is mediated by PAF [56]. PAF can activate neutrophils and induce migration of isolated neutrophils [28]. Treatment of PAF with SsE abolishes the ability of PAF to activate and induce migration of neutrophils. PAF can function as a chemoattractant in the neutrophil responses during GAS infection. It is also possible that PAF plays a role in both the inflammatory response and chemotaxis during GAS infection. PAF also activates platelets in human and some animals. However, the inhibition of the PAF-induced activation of platelets does not play a role in the phenotype of the Δ*sse* mutants in the mouse infections since murine platelets do not produce the PAF receptor according to Dr. Guy Zimmerman at University of Utah. We will examine how PAF contributes to the neutrophil response during GAS infections in our follow-up studies.

## Materials and Methods

### Declaration of Ethical Approval

All animal experimental procedures were carried out in strict accordance with the recommendations in the Guide for the Care and Use of Laboratory Animals of the National Institutes of Health. The protocols for the experiments performed at Montana State University (MSU) and Federal University of Minas Gerais (UFMG) were approved by the Institutional Animal Care and Use Committee at MSU (Permit number: 2009-09) and the Animal Ethics Committee of Instituto de Ciências Biológicas (Permit number: 168/11) (Belo Horizonte, Brazil), respectively. Blood was collected from healthy donors in accordance with a protocol approved by the Institutional Review Board at MSU (Protocol No. BL031109). Written informed consent was provided by study participants and/or their legal guardians.

### Materials

PAF (1-O-hexadecyl-2-acetyl-sn-glycero-3-phosphorylcholine), lyso-PAF C-16 (1-O-hexadecyl-sn-glycero-3-phosphocholine), human recombinant plasma PAF acetylhydrolase, heptanoyl thio-PC, and the PAF acetylhydrolase assay kit using 2-thio-PAF as the substrate were purchased from Cayman Chemical (An Harbor, MI, USA). Whatman LK6D Silica Gel 60A thin layer chromatography plates were purchased from Whatman International LLC (Clifton, NJ, USA). Recombinant wild-type and S178A mutant SsE proteins were prepared, as previously described [39].

### Bacterial Strains and Growth

MGAS5005 is a hypervirulent M1T1 GAS strain isolated from an invasive case in Ontario [9]. MGAS2221 is a M1T1 GAS strain isolated from a scarlet fever patient [48]. MGAS5005 and MGAS2221 have almost identical genetic contents but the former has a null *covS* 1-bp deletion [18]. Δ*sse* ^MGAS5005^, an in-frame *sse* deletion mutant of MGAS5005 missing amino acids 55–261 of SsE and Δ*sse-sse*, a reverse complement strain of Δ*sse*, have been described [40]. The same *sse* deletion procedure was followed to obtain Δ*sse* ^MGAS2221^. These bacteria for experiments conducted at MSU were grown to mid-exponential phase at 37°C in 5% CO_2_ in THY. GAS bacteria used in the PAFR^−/−^ experiment at UFMG were grown in brain heart infusion broth (BHI). Tryptose agar with 5% sheep blood, THY agar, and BHI agar were used as the solid media. GAS bacteria used for the animal experiments were harvested at the exponential growth phase and washed three times with and resuspended in pyrogen-free phosphate-buffered saline (PBS) to desired doses.

### Assays for PAF Acetylhydrolase Activity of SsE

SsE-catalyzed hydrolysis of PAF was monitored by TLC and LC-MS analyses and a colorimetric assay. For TLC analysis, 1.4 mM PAF was mixed with 0.08 µM wild-type SsE or SsE^S178A^ in 50 µl of 20 mM Tris-HCl, pH 8.0, and the reaction was stopped by adding 50 µl acetonitrile containing 1% formic acid after 10-min incubation at room temperature. Two µl of the reaction samples, untreated PAF, lyso-PAF, and PAF/lyso-PAF mixtures were spotted on a TLC plate, and these compounds were resolved using a methanol/chloroform/water (65∶30∶6 by volume) mixture as the mobile phase. After chromatography, PAF and lyso-PAF were visualized by spraying with 5% ammonium molybdate sulfate and heating. Protein concentrations were determined using the modified Lowry protein assay kit from Pierce with bovine serum albumin as a standard.

For LC-MS analysis, PAF hydrolysis reactions were performed as in the TLC analysis and stopped at 0 and 5 min after mixing for the wild-type SsE/PAF reaction and 40 min for the SsE^S178A^/PAF reaction. The samples were diluted with 5% acetonitrile containing 1% formic acid, and 1 µl of the diluted samples were analyzed by reverse-phase liquid chromatography and positive ion mass spectroscopy using an Agilent 1100 HPLC with autosampler (Agilent Technologies, Inc., Santa Clara, CA, USA) and a Bruker micrOTOF mass spectrometer (Bruker Daltonik GmbH, Bremen, German). The reverse-phase chromatography consisted of a 3.2-ml gradient between H_2_O and 95% acetonitrile, both with 0.1% formic acid, using a Michrom Bioresources C8 column (8×1 mm). The LC/MS data were analyzed using DataAnalysis 4.0 software (Bruker Daltonik GmbH). The mass spectrometer was calibrated using the peaks between 118 and 922 m/z of the Agilent G2421A electrospray calibrant solution infused directly to the source at a rate of 180 µl/h. PAF and lyso-PAF compounds were identified via high mass accuracy with positive control samples with m/z values of 482.3600 and 524.3722, respectively (actual masses of 482.3605 and 524.3711, errors of −1 ppm and +0.2 ppm, respectively). PAF and lyso-PAF were evaluated for carry-over on the C8 column with blank runs, but the C8 column with the described chromatography had no detectable carry-over between runs.

The colorimetric assay used the PAF acetylhydrolase assay kit from Cayman Chemical. The reactions were initiated by mixing 100 µl 20 mM Tris-HCl, pH 8.0, containing 2-thio-PAF at various concentrations and 100 µl Tris-HCl containing 4.3 nM SsE and 0.5 mM DTNB at room temperature in a 96-well plate. Absorbance at 414 nm (A_414_) of the reaction mixture was recorded every 6 s using a SPECTRA^Max^ 384 Plus spectrophotometer (Molecular Devices, Sunnyvale, CA, USA) and was used to determine initial rates of hydrolysis of 2-thio-PAF as described in the Results section.

### Isolation of Human Neutrophils

Neutrophils were isolated from the blood using dextran sedimentation, followed by Histopaque 1077 gradient separation and hypotonic lysis of red blood cells, as described previously [57]. Isolated neutrophils were washed twice and resuspended in HBSS without Ca^2+^ and Mg^2+^ for Ca^2+^ mobilization or with Ca^2+^ and Mg^2+^ for chemotaxis measurement. Neutrophil preparations were >95% pure, as determined by light microscopy, and >98% viable, as determined by trypan blue exclusion.

### Ca^2+^ Mobilization Assay

Changes in free intracellular [Ca^2+^] were measured with a FlexStation II Scanning Fluorometer (Molecular Devices) using Fluo-4 acetoxymethyl ester (Invitrogen), as previously described [58]. Briefly, human neutrophils, suspended in Hanks' balanced salt solution (HBSS) containing 10 mM HEPES, were loaded with Fluo-4 acetoxymethyl ester dye (1.25 ìg/ml final concentration) for 30 min in the dark at 37°C. After dye loading, the cells were washed with HBSS containing 10 mM HEPES, resuspended in HBSS containing 10 mM HEPES and Ca^2+^ and Mg^2+^, and separated into aliquots, which were inserted into the wells of flat-bottomed, half-area-well black microtiter plates (2×10^5^ cells/well). After addition of untreated or SsE-treated PAF, changes in fluorescence were monitored (λ_ex_ = 485 nm, λ_em_ = 538 nm) every 5 s for 240 s at room temperature.

### Chemotaxis Assay

The chemotaxis assay was performed using the ChemoTx Disposable Chemotaxis System in a 96 well microplate format (Neuro Probe, Inc., Gaithersburg, MD, USA) and the CellTitr-Glo Luminescent Cell Viability Assay (Promega, Madison, WI, USA), as described previously [58]. PAF (1.4 mM) was incubated with 0.08 µM SsE or SsE^S178A^ in 50 µl PBS, pH 7.0, at room temperature for 30 min, and the reaction was stopped by adding an equal volume of acetonitrile. Untreated and treated PAF were diluted to desired concentrations with HBSS containing 10 mM HEPES, Ca^2+^, Mg^2+^, and 0.1% BSA (HBSS/BSA). The protein control reaction samples were diluted by the same fold of the dilution as the treated PAF samples. The samples were added to wells of the assay plate at 30 µl/well in 4 replicates. The plate was covered with the filter, and 4×10^4^ neutrophils/well were placed on the top of the filter. The plate was incubated at 37°C for 1 h. Neutrophils that did not migrate were removed, and 20 µl/well of 2.5 mM EDTA was added. After incubating for 10 min at 4°C, the EDTA solution was removed, the plate was centrifuged at 600 rpm for 5 min, and 20 µl/well of CellTitr-Glo Luminescent Cell Viability Assay reagent was added. Luminescence from each well was monitored using a Fluoroscan Ascent FL Luminometer (Thermo Electron Corporation). The number of migrated cells was determined based on a standard curve using known numbers of neutrophils.

### Mouse Infections

Groups of five-week-old female inbred BALB/c and outbred CD-1 Swiss mice (Charles River Laboratory) were subcutaneously infected with 0.2 ml of an OD_600_ of 0.8 of GAS suspension in PBS or at indicated doses. Inocula were determined by plating. Mice were sacrificed at 24 h to collect skin samples for histological analyses and measurement of neutrophil infiltration and GAS CFU. Infected mice in virulence studies were monitored twice a day to get survival rates.

The PAFR^−/−^ mouse experiment was similarly performed at Dr. Mauro Teixeira's laboratory at UFMG, Brazil. BALB/c mice (8 to 12 week-old) were obtained from CEBIO (Bioterism Center) of UFMG, and PAFR^−/−^ mice (8 to 12 week-old) were generated as previously described and backcrossed at least 10 generations into a BALB/c background [30], [53]. Mice were housed in standard conditions and had free access to commercial chow and water.

### Quantification of Neutrophil Infiltration

Whole infection area in the skin was recognized by the boundary of the inflammation after the skin around the infection site was peeled off (Figure S2). The skin containing the infection area was excised and traced on a paper, which was used to measure the area of infection sites by weighing the traced paper. Numbers of recruited neutrophils in the excised skin were estimated by the myeloperoxidase assay, as described previously [44]. Skin samples were grinded in 0.5% hexadecyltrimethylammonium bromide in 50 mM potassium and sonicated on ice for 15 seconds to extract myeloperoxidase. The samples were frozen and thawed for 3 times, sonicated, and centrifuged at 16,000 g for 5 min. The myeloperoxidase activity in the supernatant obtained was measured colormetrically in 0.2 ml of 50 mM phosphate buffer, pH 6.0, containing the supernatant, 0.167 mg/ml o-dianisidine dihydrochloride, and 0.001% hydrogen peroxide. The change in absorbance at 460 nm (ΔA_460_) was recorded with time with a SPECTRA^max^ 384 Plus spectrophotometer (Molecular Devices). The myeloperoxidase activity, ΔA_460_/min, was converted into the number of neutrophils using a stand curve of myeloperoxidase activities versus known numbers of murine neutrophils, which were isolated from the bone marrow of mice, as previously described [57].

### Histological Analyses

Skin samples were excised with a wide margin around the infection site after the skin was peeled off and fixed in 10% neutral buffered formalin for 24 h. The samples were dehydrated with ethanol, cleared with xylene, and infiltrated with paraffin using a Tissue Embedding Console System (Sakura Finetek, Inc.). The paraffin blocks was processed to obtain 4-µm sections, which were stained with H&E or with a tissue Gram stain kit from Richard-Allan Scientific according to the manufacturer's protocol. The stained slides were examined using a Nikon ECLIPSE 80*i* microscope.

### GAS Clearance and Competitive Growth Assay

Clearance of GAS in the skin was measured by determining the numbers of viable GAS at infection sites. The skin around the infection sites was peeled off, excised, and grinded in 2 ml of PBS to recover bacteria, and the samples at appropriate dilution were plated on tryptose agar with 5% sheep blood to count cfu as the number of viable GAS. In the competitive growth assay, 0.2 ml of a 1∶1 Δ*sse* ^MGAS5005^∶MGAS5005 or Δ*sse* ^MGAS2221^∶MGAS2221 mixture with 0.8 ml air was injected subcutaneously into mice. The mice were euthanized at 24 h after inoculation, and the air sac was lavaged with 1 ml PBS. The lavage samples at appropriate dilution were plated on THY agar plates. The Δ*sse*/wt GAS ratio in the lavage samples was determined by analyzing 96 colonies of each sample with colony PCR using primers 5′-ATAACATTTACATTAAGGAGATAC-3′ and 5′-CAGATTTGGTGTTTGAAAAAG-3′, which yielded 1232-bp and 611-bp PCR products for the wt and Δ*sse* strains respectively. The Δ*sse*/wt GAS ratio in the inoculum was determined by plating the individual GAS suspension prior to mixing. The competitive index is calculated by dividing the Δ*sse*/wt GAS ratio in the lavage samples by the ratio in the inoculum.

### In Trans Overexpression of SsE

The *sse* gene of MGAS5005 was PCR cloned into pDCBB [45] at the XbaI and EcoRI sites using primers 5′-ATCTAGAATAACATTTACATTAAGGAGATAC-3′ and 5′-AGAATTCCAGATTTGGTGTTT-3′, yielding pSsE. MGAS2221 was transformed with pSsE for in trans SsE overexpression and with pDCBB for vector control. Levels of SsE in the culture supernatant of MGAS2221/pDCBB and MGAS2221/pSsE were compared using Western blotting, as previously described [39].

### Statistic Analyses

Statistic analyses of the data of the animal experiments were performed using the GraphPad Prism software with the following tests: Log-rank (Mantel-Cox) Test for the survival data in Figure 7A; one way ANOVA Tukey's Multiple Comparison Test for the data in Figure 6; and one-tailed, unpaired t test for Figures 7C, 7D, 7F, 7G, and 8D.

## Supporting Information

Figure S1 **Kinetic analysis of SsE-catalyzed hydrolysis of 2-thio-PAF.** (**A**) Time course of absorbance change at A_414_ after mixing 3.4 nM SsE with 2-thio-PAF at 10, 20, 30, 40, 50, 60, 70, 110, and 200 µM, which correspond to the curves from bottom to top. (**B**) Linear regression of the ΔA_414_ data up to the 0.6-min time point in panel A to obtain initial rates in hydrolysis of 2-thio-PAF. The rate of the hydrolysis reaction was fast, and 2-thio-PAF was consumed rapidly, even when nM of SsE was used. Thus, the absorbance data in the first 36 s of the reaction was used to calculate initial reaction rates at different 2-thio-PAF concentrations for Figure 3B. Because significant portions of the substrate had been hydrolyzed when the measurement started at time zero, we corrected substrate concentrations for Figure 3B by subtracting the hydrolyzed amounts from the total added substrate concentrations using the A_414_ readings at time zero and ε_414_ of 7.16 mM^−1^ for a light path of 0.53 cm under the assay conditions.
(TIF)

Figure S2 **Inside-out images of the MGAS5005 and Δ** ***sse*** **^MGAS5005^ infection site.** BALB/c mice were subcutaneously inoculated on the back with 1.0×10^8^ cfu MGAS5005 or 1.1×10^8^ cfu Δ*sse* ^MGAS5005^, and the skin around the infection site was collected at 24 h after inoculation. (**A**) Infection site of MGAS5005. GAS spread in the skin from the inoculation site, which is circled, toward the stomach area, and the spread area indicated by the arrow was inflamed and red in color. (**B**) Infection site of Δ*sse* ^MGAS5005^. The *sse* deletion mutant did not substantially invade the surrounding skin tissue.
(TIF)

Figure S3 **Histological images of the MGAS5005 and Δ** ***sse*** **^MGAS5005^ infection site.** BALB/c mice were subcutaneously inoculated on the back with 1.0×10^8^ cfu MGAS5005 or 1.1×10^8^ cfu Δ*sse* ^MGAS5005^, and the skin samples were collected 24 h post-inoculation. (A and B) Images of H&E (A)- and Gram (B)-stained dissection of a part of the MGAS5005 site. (C and D) Images of H&E (C)- and Gram (D)-stained dissection of the whole Δ*sse* ^MGAS5005^ infection site. The images were each combined from three snapshots that were taken at a 4× magnification. Scale bar: 500 µm. The boxes indicate the loci that are shown in Figure 5 at a 40× magnification.
(TIF)

Figure S4 **No detrimental effect of the** ***sse*** **deletion on expression of virulence genes.** The expression levels of Mga and CovRS/CsrRS regulons and the *gyrA* gene were assessed by microarray analysis using NimbleExpress *Streptococcus pyogenes* MGAS5005 arrays, as we previously described (Liu M, et al. Microbiology 152: 967–978). Because of limited resources, no replicates were performed. Presented are fluorescence intensities of the genes that were normalized with per chip per gene median polishing.
(TIF)
