# Supplementary figures and images for: Group A Streptococcus Secreted Esterase Hydrolyzes Platelet-Activating Factor to Impede Neutrophil Recruitment and Facilitate Innate Immune Evasion
Source: PLoS Pathog. 2012 Apr 5;8(4):e1002624. doi: 10.1371/journal.ppat.1002624 (PMC3320582; doi:10.1371/journal.ppat.1002624)

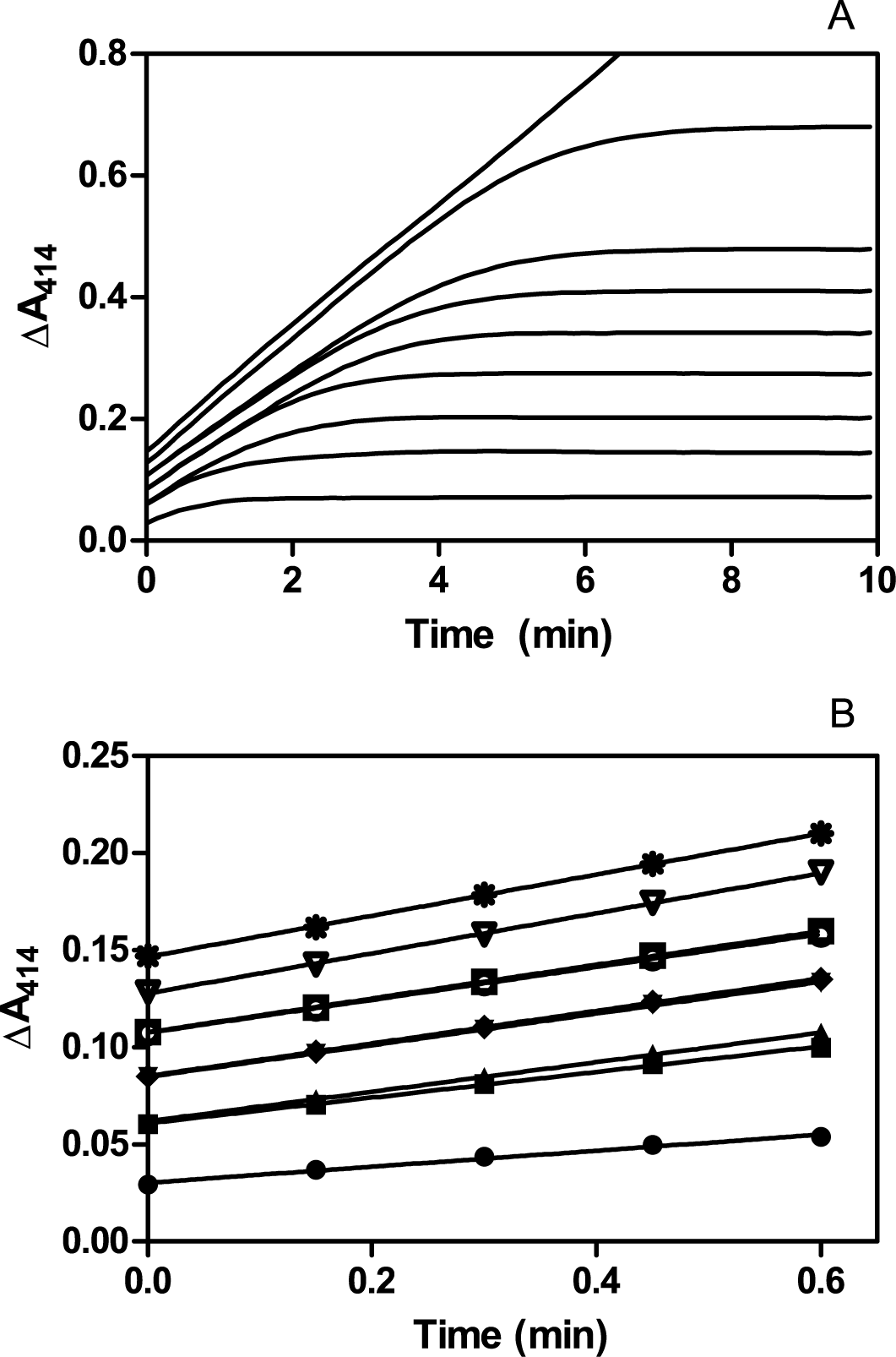

Supplement: Figure S1 — Kinetic analysis of SsE-catalyzed hydrolysis of 2-thio-PAF. (A) Time course of absorbance change at A414 after mixing 3.4 nM SsE with 2-thio-PAF at 10, 20, 30, 40, 50, 60, 70, 110, and 200 µM, which correspond to the curves from bottom to top. (B) Linear regression of the ΔA414 data up to the 0.6-min time point in panel A to obtain initial rates in hydrolysis of 2-thio-PAF. The rate of the hydrolysis reaction was fast, and 2-thio-PAF was consumed rapidly, even when nM of SsE was used. Thus, the absorbance data in the first 36 s of the reaction was used to calculate initial reaction rates at different 2-thio-PAF concentrations for Figure 3B. Because significant portions of the substrate had been hydrolyzed when the measurement started at time zero, we corrected substrate concentrations for Figure 3B by subtracting the hydrolyzed amounts from the total added substrate concentrations using the A414 readings at time zero and ε414 of 7.16 mM−1 for a light path of 0.53 cm under the assay conditions. (TIF) [file ppat.1002624.s001.tif]

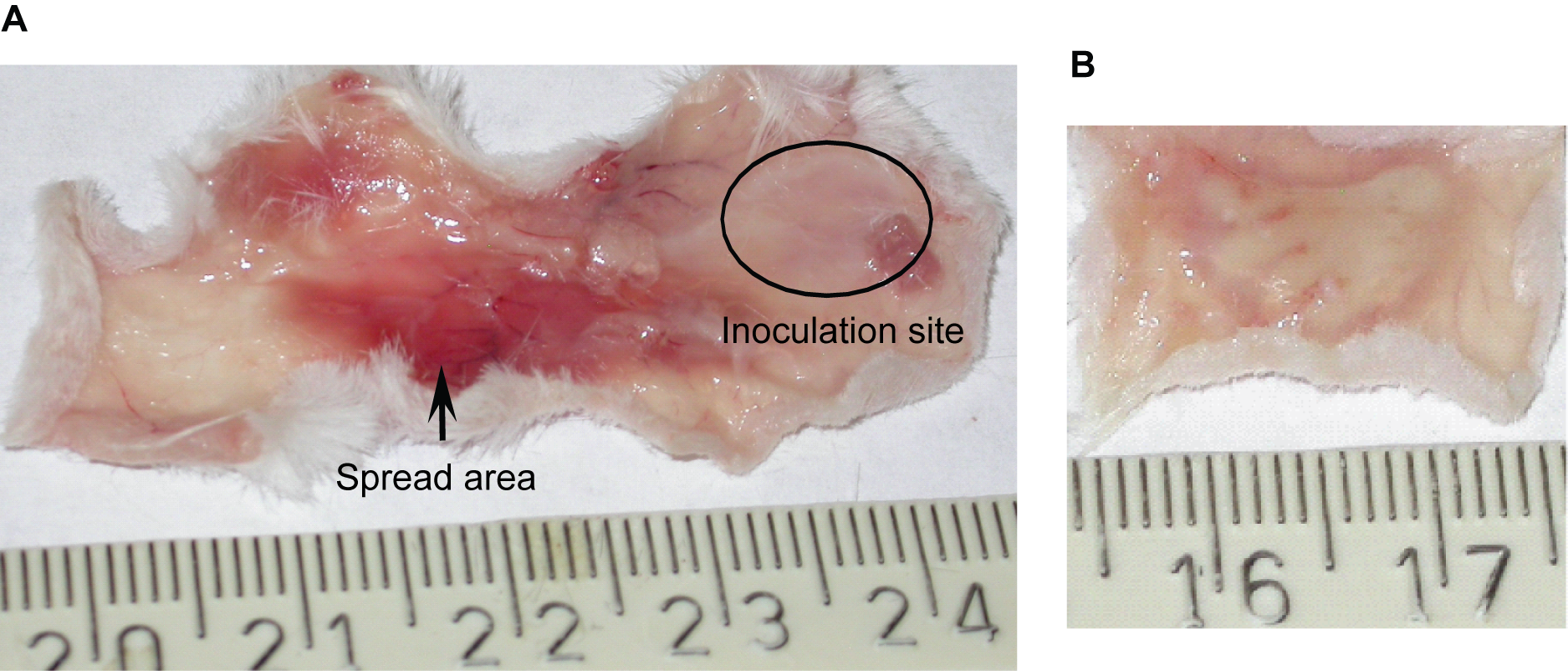

Supplement: Figure S2 — Inside-out images of the MGAS5005 and Δ sse MGAS5005 infection site. BALB/c mice were subcutaneously inoculated on the back with 1.0×10^8^ cfu MGAS5005 or 1.1×10^8^ cfu Δsse MGAS5005, and the skin around the infection site was collected at 24 h after inoculation. (A) Infection site of MGAS5005. GAS spread in the skin from the inoculation site, which is circled, toward the stomach area, and the spread area indicated by the arrow was inflamed and red in color. (B) Infection site of Δsse MGAS5005. The sse deletion mutant did not substantially invade the surrounding skin tissue. (TIF) [file ppat.1002624.s002.tif]

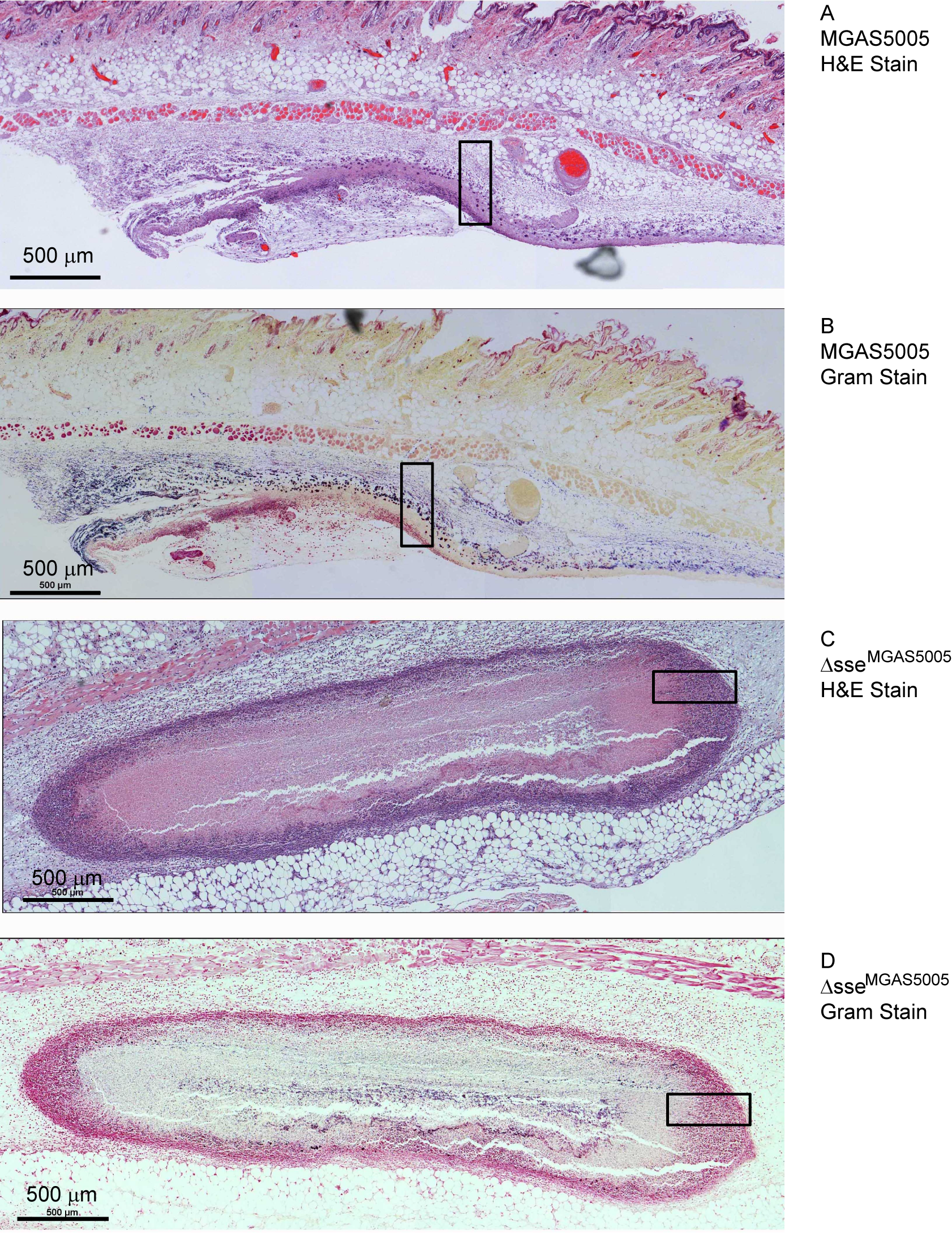

Supplement: Figure S3 — Histological images of the MGAS5005 and Δ sse MGAS5005 infection site. BALB/c mice were subcutaneously inoculated on the back with 1.0×10^8^ cfu MGAS5005 or 1.1×10^8^ cfu Δsse MGAS5005, and the skin samples were collected 24 h post-inoculation. (A and B) Images of H&E (A)- and Gram (B)-stained dissection of a part of the MGAS5005 site. (C and D) Images of H&E (C)- and Gram (D)-stained dissection of the whole Δsse MGAS5005 infection site. The images were each combined from three snapshots that were taken at a 4× magnification. Scale bar: 500 µm. The boxes indicate the loci that are shown in Figure 5 at a 40× magnification. (TIF) [file ppat.1002624.s003.tif]

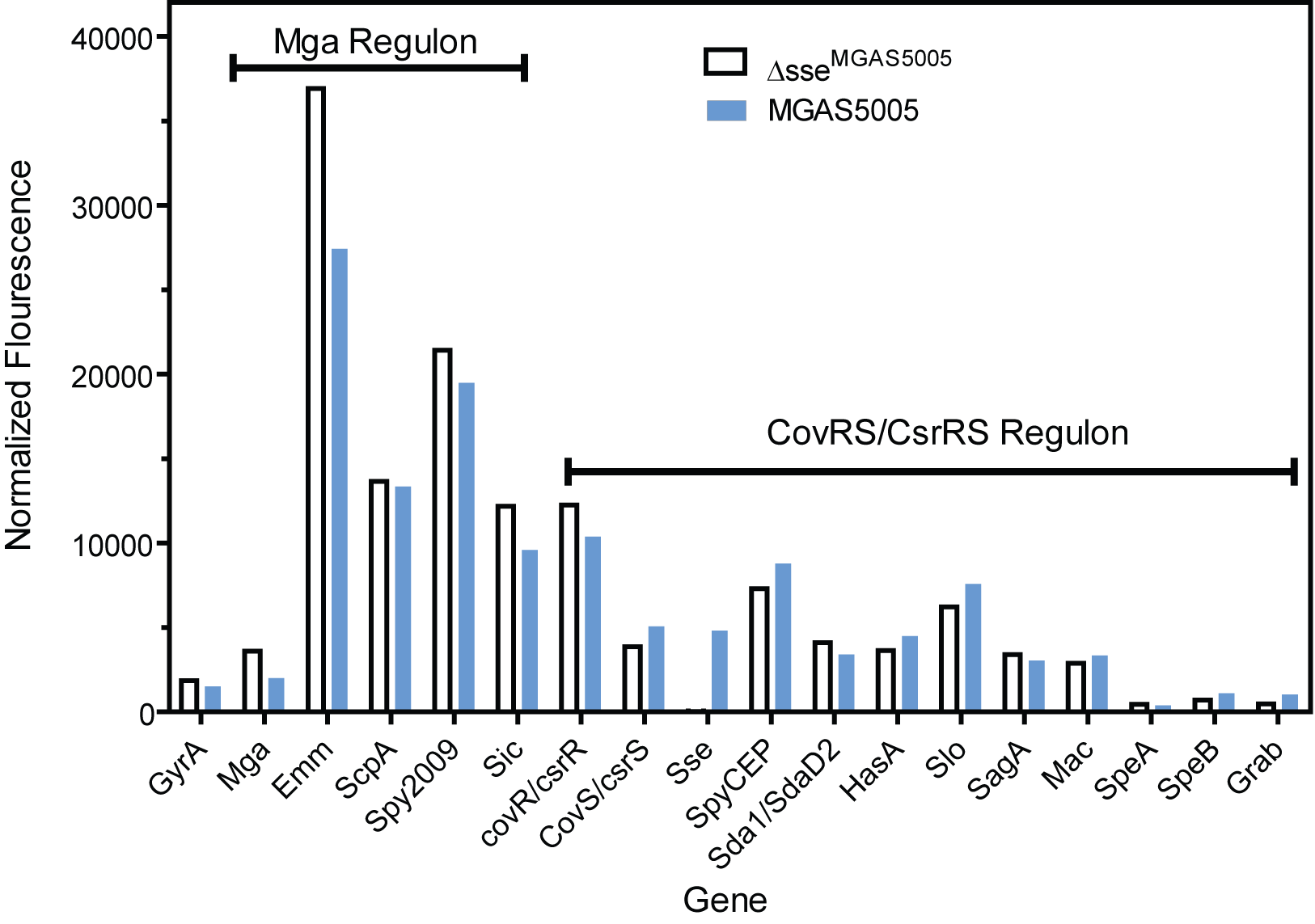

Supplement: Figure S4 — No detrimental effect of the sse deletion on expression of virulence genes. The expression levels of Mga and CovRS/CsrRS regulons and the gyrA gene were assessed by microarray analysis using NimbleExpress Streptococcus pyogenes MGAS5005 arrays, as we previously described (Liu M, et al. Microbiology 152: 967–978). Because of limited resources, no replicates were performed. Presented are fluorescence intensities of the genes that were normalized with per chip per gene median polishing. (TIF) [file ppat.1002624.s004.tif]
